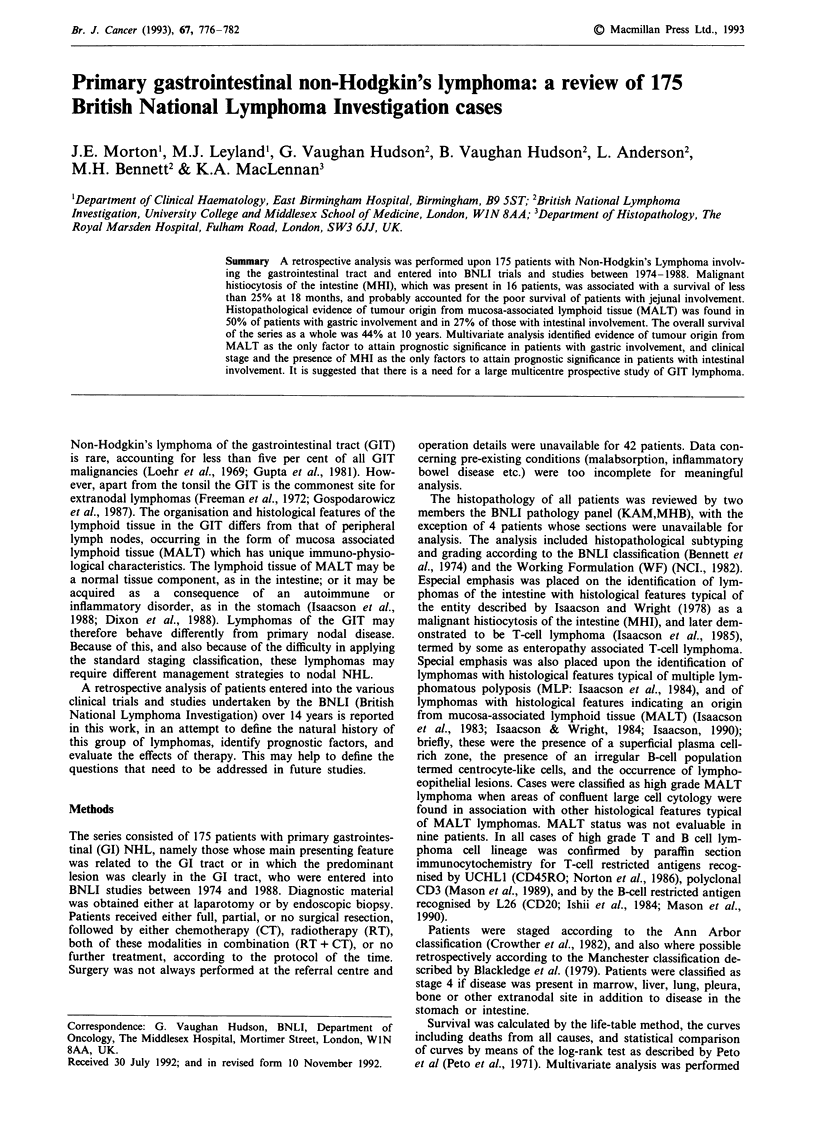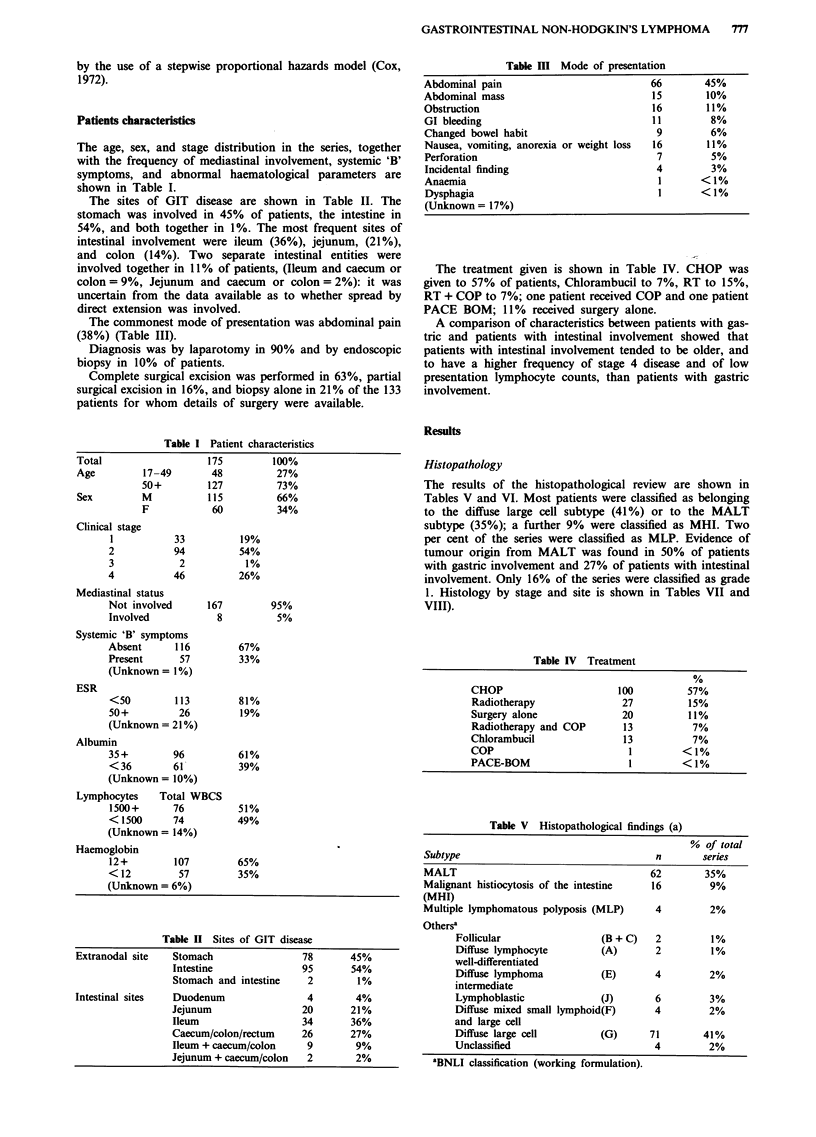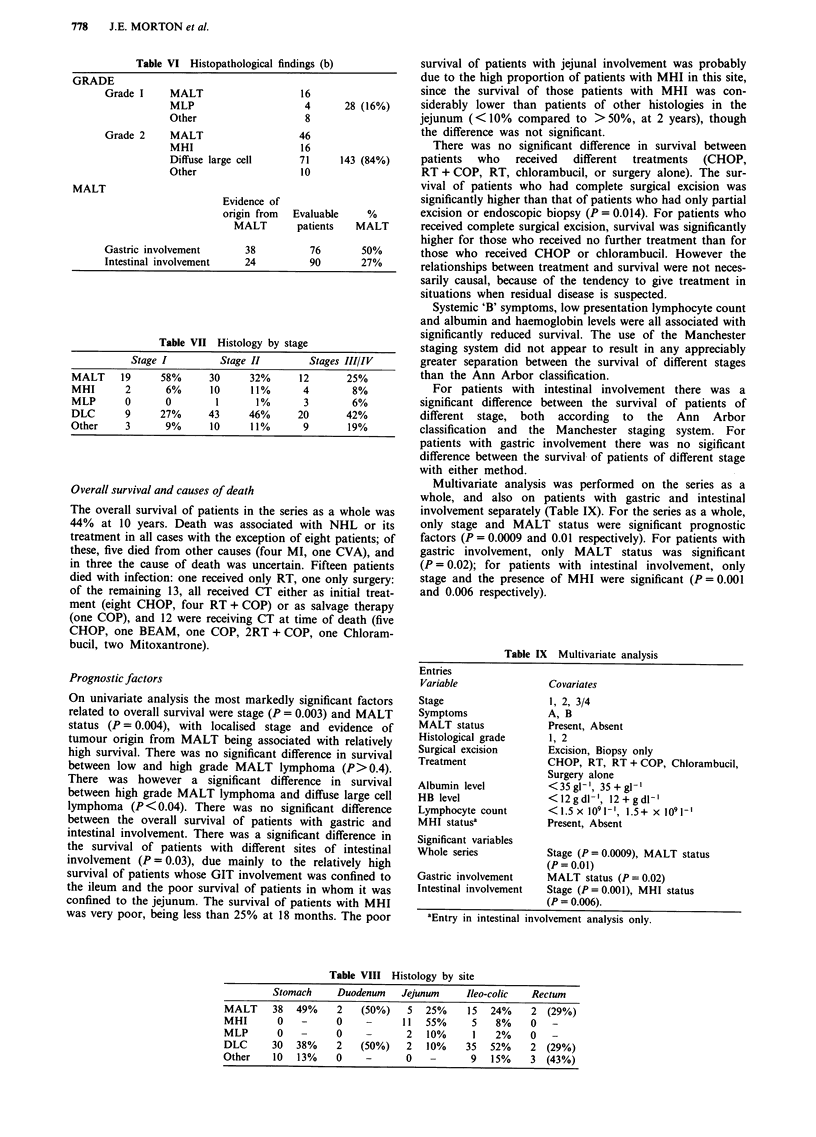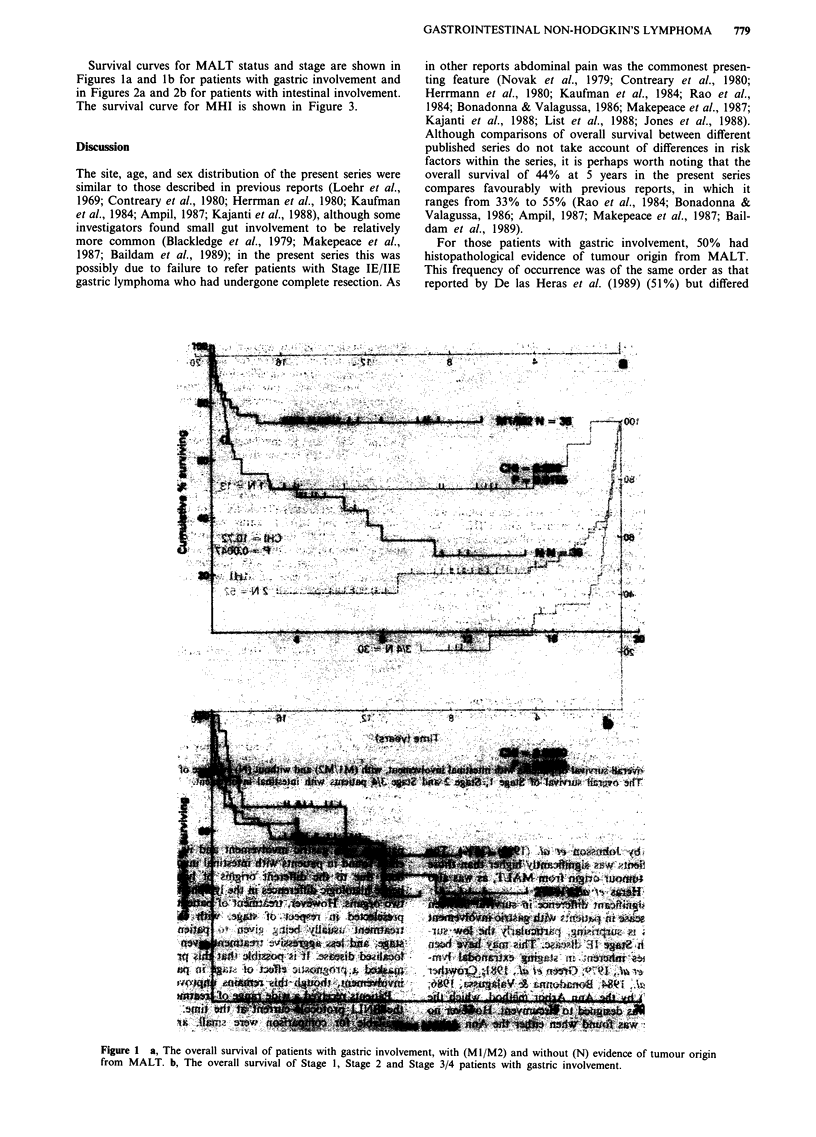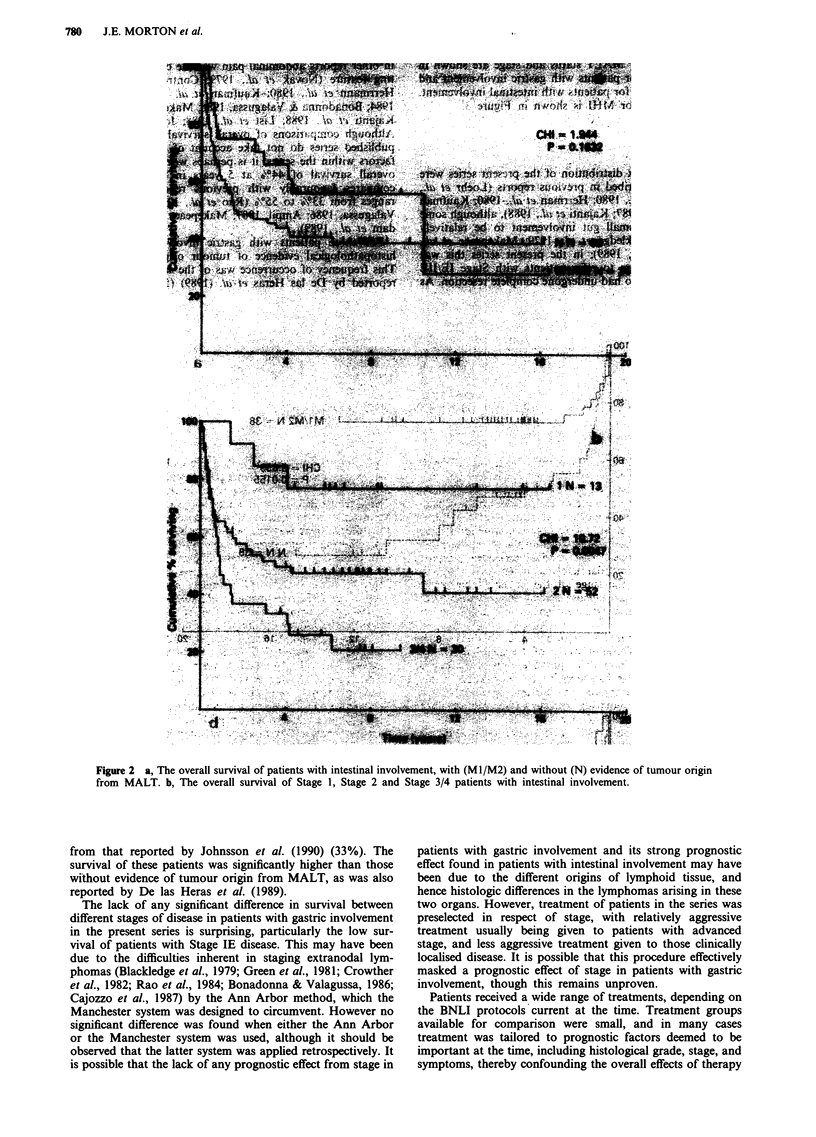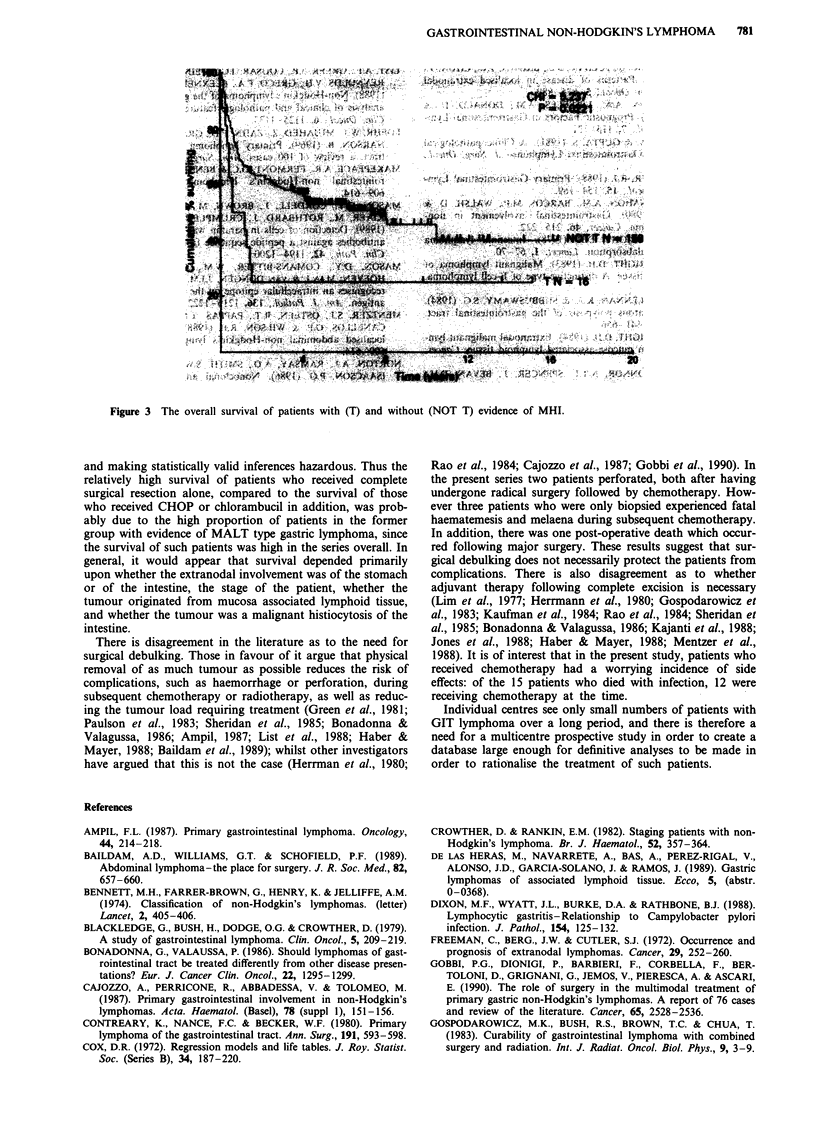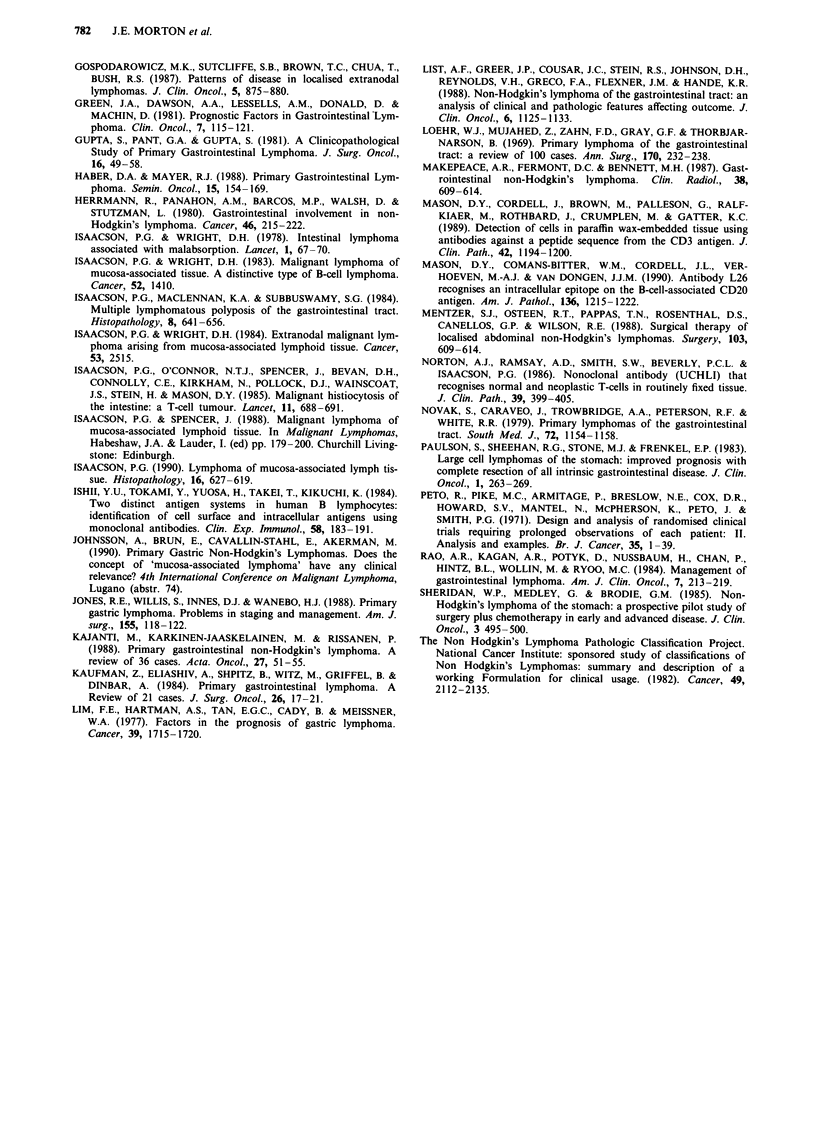# Primary gastrointestinal non-Hodgkin's lymphoma: a review of 175 British National Lymphoma Investigation cases.

**DOI:** 10.1038/bjc.1993.141

**Published:** 1993-04

**Authors:** J. E. Morton, M. J. Leyland, G. Vaughan Hudson, B. Vaughan Hudson, L. Anderson, M. H. Bennett, K. A. MacLennan

**Affiliations:** Department of Clinical Haematology, East Birmingham Hospital, UK.

## Abstract

A retrospective analysis was performed upon 175 patients with Non-Hodgkin's Lymphoma involving the gastrointestinal tract and entered into BNLI trials and studies between 1974-1988. Malignant histiocytosis of the intestine (MHI), which was present in 16 patients, was associated with a survival of less than 25% at 18 months, and probably accounted for the poor survival of patients with jejunal involvement. Histopathological evidence of tumour origin from mucosa-associated lymphoid tissue (MALT) was found in 50% of patients with gastric involvement and in 27% of those with intestinal involvement. The overall survival of the series as a whole was 44% at 10 years. Multivariate analysis identified evidence of tumour origin from MALT as the only factor to attain prognostic significance in patients with gastric involvement, and clinical stage and the presence of MHI as the only factors to attain prognostic significance in patients with intestinal involvement. It is suggested that there is a need for a large multicentre prospective study of GIT lymphoma.


Br. J. Cancer (1993), 67, 776–782 © Macmillan Press Ltd., 1993

### Primary gastrointestinal non-Hodgkin's lymphoma: a review of 175 British National Lymphoma Investigation cases

J.E. Morton[1], M.J. Leyland[1], G. Vaughan Hudson[2], B. Vaughan Hudson[2], L. Anderson[2], M.H. Bennett[2] & K.A. MacLennan[3]

[1]*Department of Clinical Haematology, East Birmingham Hospital, Birmingham, B9 5ST;* [2]*British National Lymphoma Investigation, University College and Middlesex School of Medicine, London, W1N 8AA;* [3]*Department of Histopathology, The Royal Marsden Hospital, Fulham Road, London, SW3 6JJ, UK.*

**Summary** A retrospective analysis was performed upon 175 patients with Non-Hodgkin's Lymphoma involving the gastrointestinal tract and entered into BNLI trials and studies between 1974–1988. Malignant histiocytosis of the intestine (MHI), which was present in 16 patients, was associated with a survival of less than 25% at 18 months, and probably accounted for the poor survival of patients with jejunal involvement. Histopathological evidence of tumour origin from mucosa-associated lymphoid tissue (MALT) was found in 50% of patients with gastric involvement and in 27% of those with intestinal involvement. The overall survival of the series as a whole was 44% at 10 years. Multivariate analysis identified evidence of tumour origin from MALT as the only factor to attain prognostic significance in patients with gastric involvement, and clinical stage and the presence of MHI as the only factors to attain prognostic significance in patients with intestinal involvement. It is suggested that there is a need for a large multicentre prospective study of GIT lymphoma.

Non-Hodgkin's lymphoma of the gastrointestinal tract (GIT) is rare, accounting for less than five per cent of all GIT malignancies (Loehr *et al.*, 1969; Gupta *et al.*, 1981). However, apart from the tonsil the GIT is the commonest site for extranodal lymphomas (Freeman *et al.*, 1972; Gospodarowicz *et al.*, 1987). The organisation and histological features of the lymphoid tissue in the GIT differs from that of peripheral lymph nodes, occurring in the form of mucosa associated lymphoid tissue (MALT) which has unique immuno-physiological characteristics. The lymphoid tissue of MALT may be a normal tissue component, as in the intestine; or it may be acquired as a consequence of an autoimmune or inflammatory disorder, as in the stomach (Isaacson *et al.*, 1988; Dixon *et al.*, 1988). Lymphomas of the GIT may therefore behave differently from primary nodal disease. Because of this, and also because of the difficulty in applying the standard staging classification, these lymphomas may require different management strategies to nodal NHL.

A retrospective analysis of patients entered into the various clinical trials and studies undertaken by the BNLI (British National Lymphoma Investigation) over 14 years is reported in this work, in an attempt to define the natural history of this group of lymphomas, identify prognostic factors, and evaluate the effects of therapy. This may help to define the questions that need to be addressed in future studies.

### Methods

The series consisted of 175 patients with primary gastrointestinal (GI) NHL, namely those whose main presenting feature was related to the GI tract or in which the predominant lesion was clearly in the GI tract, who were entered into BNLI studies between 1974 and 1988. Diagnostic material was obtained either at laparotomy or by endoscopic biopsy. Patients received either full, partial, or no surgical resection, followed by either chemotherapy (CT), radiotherapy (RT), both of these modalities in combination (RT + CT), or no further treatment, according to the protocol of the time. Surgery was not always performed at the referral centre and operation details were unavailable for 42 patients. Data concerning pre-existing conditions (malabsorption, inflammatory bowel disease etc.) were too incomplete for meaningful analysis.

The histopathology of all patients was reviewed by two members the BNLI pathology panel (KAM,MHB), with the exception of 4 patients whose sections were unavailable for analysis. The analysis included histopathological subtyping and grading according to the BNLI classification (Bennett *et al.*, 1974) and the Working Formulation (WF) (NCI., 1982). Especial emphasis was placed on the identification of lymphomas of the intestine with histological features typical of the entity described by Isaacson and Wright (1978) as a malignant histiocytosis of the intestine (MHI), and later demonstrated to be T-cell lymphoma (Isaacson *et al.*, 1985), termed by some as enteropathy associated T-cell lymphoma. Special emphasis was also placed upon the identification of lymphomas with histological features typical of multiple lymphomatous polyposis (MLP: Isaacson *et al.*, 1984), and of lymphomas with histological features indicating an origin from mucosa-associated lymphoid tissue (MALT) (Isaacson *et al.*, 1983; Isaacson & Wright, 1984; Isaacson, 1990); briefly, these were the presence of a superficial plasma cell-rich zone, the presence of an irregular B-cell population termed centrocyte-like cells, and the occurrence of lympho-eopithelial lesions. Cases were classified as high grade MALT lymphoma when areas of confluent large cell cytology were found in association with other histological features typical of MALT lymphomas. MALT status was not evaluable in nine patients. In all cases of high grade T and B cell lymphoma cell lineage was confirmed by paraffin section immunocytochemistry for T-cell restricted antigens recognised by UCHL1 (CD45RO; Norton *et al.*, 1986), polyclonal CD3 (Mason *et al.*, 1989), and by the B-cell restricted antigen recognised by L26 (CD20; Ishii *et al.*, 1984; Mason *et al.*, 1990).

Patients were staged according to the Ann Arbor classification (Crowther *et al.*, 1982), and also where possible retrospectively according to the Manchester classification described by Blackledge *et al.* (1979). Patients were classified as stage 4 if disease was present in marrow, liver, lung, pleura, bone or other extranodal site in addition to disease in the stomach or intestine.

Survival was calculated by the life-table method, the curves including deaths from all causes, and statistical comparison of curves by means of the log-rank test as described by Peto *et al* (Peto *et al.*, 1971). Multivariate analysis was performed

Correspondence: G. Vaughan Hudson, BNLI, Department of Oncology, The Middlesex Hospital, Mortimer Street, London, W1N 8AA, UK.
Received 30 July 1992; and in revised form 10 November 1992.

by the use of a stepwise proportional hazards model (Cox, 1972).

**Patients characteristics**

The age, sex, and stage distribution in the series, together with the frequency of mediastinal involvement, systemic 'B' symptoms, and abnormal haematological parameters are shown in Table I.

The sites of GIT disease are shown in Table II. The stomach was involved in 45% of patients, the intestine in 54%, and both together in 1%. The most frequent sites of intestinal involvement were ileum (36%), jejunum, (21%), and colon (14%). Two separate intestinal entities were involved together in 11% of patients, (Ileum and caecum or colon = 9%, Jejunum and caecum or colon = 2%): it was uncertain from the data available as to whether spread by direct extension was involved.

The commonest mode of presentation was abdominal pain (38%) (Table III).

Diagnosis was by laparotomy in 90% and by endoscopic biopsy in 10% of patients.

Complete surgical excision was performed in 63%, partial surgical excision in 16%, and biopsy alone in 21% of the 133 patients for whom details of surgery were available.

**Table I** Patient characteristics

| | | | | |
|---|---|---|---|---|
| Total | | 175 | | 100% |
| Age | 17–49 | 48 | | 27% |
| | 50+ | 127 | | 73% |
| Sex | M | 115 | | 66% |
| | F | 60 | | 34% |
| Clinical stage | | | | |
| 1 | 33 | | 19% | |
| 2 | 94 | | 54% | |
| 3 | 2 | | 1% | |
| 4 | 46 | | 26% | |
| Mediastinal status | | | | |
| Not involved | | 167 | | 95% |
| Involved | | 8 | | 5% |
| Systemic 'B' symptoms | | | | |
| Absent | 116 | | 67% | |
| Present | 57 | | 33% | |
| (Unknown = 1%) | | | | |
| ESR | | | | |
| <50 | 113 | | 81% | |
| 50+ | 26 | | 19% | |
| (Unknown = 21%) | | | | |
| Albumin | | | | |
| 35+ | 96 | | 61% | |
| <36 | 61 | | 39% | |
| (Unknown = 10%) | | | | |
| Lymphocytes | Total WBCS | | | |
| 1500+ | 76 | | 51% | |
| <1500 | 74 | | 49% | |
| (Unknown = 14%) | | | | |
| Haemoglobin | | | | |
| 12+ | 107 | | 65% | |
| <12 | 57 | | 35% | |
| (Unknown = 6%) | | | | |

**Table II** Sites of GIT disease

| | | | |
|---|---|---|---|
| Extranodal site | Stomach | 78 | 45% |
| | Intestine | 95 | 54% |
| | Stomach and intestine | 2 | 1% |
| Intestinal sites | Duodenum | 4 | 4% |
| | Jejunum | 20 | 21% |
| | Ileum | 34 | 36% |
| | Caecum/colon/rectum | 26 | 27% |
| | Ileum + caecum/colon | 9 | 9% |
| | Jejunum + caecum/colon | 2 | 2% |

**Table III** Mode of presentation

| | | |
|---|---|---|
| Abdominal pain | 66 | 45% |
| Abdominal mass | 15 | 10% |
| Obstruction | 16 | 11% |
| GI bleeding | 11 | 8% |
| Changed bowel habit | 9 | 6% |
| Nausea, vomiting, anorexia or weight loss | 16 | 11% |
| Perforation | 7 | 5% |
| Incidental finding | 4 | 3% |
| Anaemia | 1 | <1% |
| Dysphagia | 1 | <1% |
| (Unknown = 17%) | | |

The treatment given is shown in Table IV. CHOP was given to 57% of patients, Chlorambucil to 7%, RT to 15%, RT + COP to 7%; one patient received COP and one patient PACE BOM; 11% received surgery alone.

A comparison of characteristics between patients with gastric and patients with intestinal involvement showed that patients with intestinal involvement tended to be older, and to have a higher frequency of stage 4 disease and of low presentation lymphocyte counts, than patients with gastric involvement.

**Results**

*Histopathology*

The results of the histopathological review are shown in Tables V and VI. Most patients were classified as belonging to the diffuse large cell subtype (41%) or to the MALT subtype (35%); a further 9% were classified as MHI. Two per cent of the series were classified as MLP. Evidence of tumour origin from MALT was found in 50% of patients with gastric involvement and 27% of patients with intestinal involvement. Only 16% of the series were classified as grade 1. Histology by stage and site is shown in Tables VII and VIII).

**Table IV** Treatment

| | | % |
|---|---|---|
| CHOP | 100 | 57% |
| Radiotherapy | 27 | 15% |
| Surgery alone | 20 | 11% |
| Radiotherapy and COP | 13 | 7% |
| Chlorambucil | 13 | 7% |
| COP | 1 | <1% |
| PACE-BOM | 1 | <1% |

**Table V** Histopathological findings (a)

| *Subtype* | | *n* | *% of total series* |
|---|---|---|---|
| MALT | | 62 | 35% |
| Malignant histiocytosis of the intestine (MHI) | | 16 | 9% |
| Multiple lymphomatous polyposis (MLP) | | 4 | 2% |
| Others[a] | | | |
| Follicular | (B + C) | 2 | 1% |
| Diffuse lymphocyte well-differentiated | (A) | 2 | 1% |
| Diffuse lymphoma intermediate | (E) | 4 | 2% |
| Lymphoblastic | (J) | 6 | 3% |
| Diffuse mixed small lymphoid and large cell | (F) | 4 | 2% |
| Diffuse large cell | (G) | 71 | 41% |
| Unclassified | | 4 | 2% |

[a]BNLI classification (working formulation).

**Table VI** Histopathological findings (b)

| | | | |
|---|---|---|---|
| GRADE | | | |
| Grade I | MALT | 16 | |
| | MLP | 4 | 28 (16%) |
| | Other | 8 | |
| Grade 2 | MALT | 46 | |
| | MHI | 16 | |
| | Diffuse large cell | 71 | 143 (84%) |
| | Other | 10 | |

| MALT | Evidence of origin from MALT | Evaluable patients | % MALT |
|---|---|---|---|
| Gastric involvement | 38 | 76 | 50% |
| Intestinal involvement | 24 | 90 | 27% |

**Table VII** Histology by stage

| | *Stage I* | | *Stage II* | | *Stages III/IV* | |
|---|---|---|---|---|---|---|
| MALT | 19 | 58% | 30 | 32% | 12 | 25% |
| MHI | 2 | 6% | 10 | 11% | 4 | 8% |
| MLP | 0 | 0 | 1 | 1% | 3 | 6% |
| DLC | 9 | 27% | 43 | 46% | 20 | 42% |
| Other | 3 | 9% | 10 | 11% | 9 | 19% |

*Overall survival and causes of death*

The overall survival of patients in the series as a whole was 44% at 10 years. Death was associated with NHL or its treatment in all cases with the exception of eight patients; of these, five died from other causes (four MI, one CVA), and in three the cause of death was uncertain. Fifteen patients died with infection: one received only RT, one only surgery: of the remaining 13, all received CT either as initial treatment (eight CHOP, four RT + COP) or as salvage therapy (one COP), and 12 were receiving CT at time of death (five CHOP, one BEAM, one COP, 2RT + COP, one Chlorambucil, two Mitoxantrone).

*Prognostic factors*

On univariate analysis the most markedly significant factors related to overall survival were stage ($P = 0.003$) and MALT status ($P = 0.004$), with localised stage and evidence of tumour origin from MALT being associated with relatively high survival. There was no significant difference in survival between low and high grade MALT lymphoma ($P > 0.4$). There was however a significant difference in survival between high grade MALT lymphoma and diffuse large cell lymphoma ($P < 0.04$). There was no significant difference between the overall survival of patients with gastric and intestinal involvement. There was a significant difference in the survival of patients with different sites of intestinal involvement ($P = 0.03$), due mainly to the relatively high survival of patients whose GIT involvement was confined to the ileum and the poor survival of patients in whom it was confined to the jejunum. The survival of patients with MHI was very poor, being less than 25% at 18 months. The poor survival of patients with jejunal involvement was probably due to the high proportion of patients with MHI in this site, since the survival of those patients with MHI was considerably lower than patients of other histologies in the jejunum (<10% compared to >50%, at 2 years), though the difference was not significant.

There was no significant difference in survival between patients who received different treatments (CHOP, RT + COP, RT, chlorambucil, or surgery alone). The survival of patients who had complete surgical excision was significantly higher than that of patients who had only partial excision or endoscopic biopsy ($P = 0.014$). For patients who received complete surgical excision, survival was significantly higher for those who received no further treatment than for those who received CHOP or chlorambucil. However the relationships between treatment and survival were not necessarily causal, because of the tendency to give treatment in situations when residual disease is suspected.

Systemic 'B' symptoms, low presentation lymphocyte count and albumin and haemoglobin levels were all associated with significantly reduced survival. The use of the Manchester staging system did not appear to result in any appreciably greater separation between the survival of different stages than the Ann Arbor classification.

For patients with intestinal involvement there was a significant difference between the survival of patients of different stage, both according to the Ann Arbor classification and the Manchester staging system. For patients with gastric involvement there was no sigificant difference between the survival of patients of different stage with either method.

Multivariate analysis was performed on the series as a whole, and also on patients with gastric and intestinal involvement separately (Table IX). For the series as a whole, only stage and MALT status were significant prognostic factors ($P = 0.0009$ and 0.01 respectively). For patients with gastric involvement, only MALT status was significant ($P = 0.02$); for patients with intestinal involvement, only stage and the presence of MHI were significant ($P = 0.001$ and 0.006 respectively).

**Table IX** Multivariate analysis

| Entries *Variable* | *Covariates* |
|---|---|
| Stage | 1, 2, 3/4 |
| Symptoms | A, B |
| MALT status | Present, Absent |
| Histological grade | 1, 2 |
| Surgical excision | Excision, Biopsy only |
| Treatment | CHOP, RT, RT + COP, Chlorambucil, Surgery alone |
| Albumin level | $<35\,gl^{-1}$, $35+gl^{-1}$ |
| HB level | $<12\,g\,dl^{-1}$, $12+g\,dl^{-1}$ |
| Lymphocyte count | $<1.5\times10^{9}\,l^{-1}$, $1.5+\times10^{9}\,l^{-1}$ |
| MHI status[a] | Present, Absent |
| Significant variables | |
| Whole series | Stage ($P = 0.0009$), MALT status ($P = 0.01$) |
| Gastric involvement | MALT status ($P = 0.02$) |
| Intestinal involvement | Stage ($P = 0.001$), MHI status ($P = 0.006$). |

[a]Entry in intestinal involvement analysis only.

**Table VIII** Histology by site

| | *Stomach* | | *Duodenum* | | *Jejunum* | | *Ileo-colic* | | *Rectum* | |
|---|---|---|---|---|---|---|---|---|---|---|
| MALT | 38 | 49% | 2 | (50%) | 5 | 25% | 15 | 24% | 2 | (29%) |
| MHI | 0 | – | 0 | – | 11 | 55% | 5 | 8% | 0 | – |
| MLP | 0 | – | 0 | – | 2 | 10% | 1 | 2% | 0 | – |
| DLC | 30 | 38% | 2 | (50%) | 2 | 10% | 35 | 52% | 2 | (29%) |
| Other | 10 | 13% | 0 | – | 0 | – | 9 | 15% | 3 | (43%) |

Survival curves for MALT status and stage are shown in Figures 1a and 1b for patients with gastric involvement and in Figures 2a and 2b for patients with intestinal involvement. The survival curve for MHI is shown in Figure 3.

### Discussion

The site, age, and sex distribution of the present series were similar to those described in previous reports (Loehr *et al.*, 1969; Contreary *et al.*, 1980; Herrman *et al.*, 1980; Kaufman *et al.*, 1984; Ampil, 1987; Kajanti *et al.*, 1988), although some investigators found small gut involvement to be relatively more common (Blackledge *et al.*, 1979; Makepeace *et al.*, 1987; Baildam *et al.*, 1989); in the present series this was possibly due to failure to refer patients with Stage IE/IIE gastric lymphoma who had undergone complete resection. As in other reports abdominal pain was the commonest presenting feature (Novak *et al.*, 1979; Contreary *et al.*, 1980; Herrmann *et al.*, 1980; Kaufman *et al.*, 1984; Rao *et al.*, 1984; Bonadonna & Valagussa, 1986; Makepeace *et al.*, 1987; Kajanti *et al.*, 1988; List *et al.*, 1988; Jones *et al.*, 1988). Although comparisons of overall survival between different published series do not take account of differences in risk factors within the series, it is perhaps worth noting that the overall survival of 44% at 5 years in the present series compares favourably with previous reports, in which it ranges from 33% to 55% (Rao *et al.*, 1984; Bonadonna & Valagussa, 1986; Ampil, 1987; Makepeace *et al.*, 1987; Baildam *et al.*, 1989).

For those patients with gastric involvement, 50% had histopathological evidence of tumour origin from MALT. This frequency of occurrence was of the same order as that reported by De las Heras *et al.* (1989) (51%) but differed

**Figure 1** **a**, The overall survival of patients with gastric involvement, with (M1/M2) and without (N) evidence of tumour origin from MALT. **b**, The overall survival of Stage 1, Stage 2 and Stage 3/4 patients with gastric involvement.

**Figure 2** **a**, The overall survival of patients with intestinal involvement, with (M1/M2) and without (N) evidence of tumour origin from MALT. **b**, The overall survival of Stage 1, Stage 2 and Stage 3/4 patients with intestinal involvement.

from that reported by Johnsson *et al.* (1990) (33%). The survival of these patients was significantly higher than those without evidence of tumour origin from MALT, as was also reported by De las Heras *et al.* (1989).

The lack of any significant difference in survival between different stages of disease in patients with gastric involvement in the present series is surprising, particularly the low survival of patients with Stage IE disease. This may have been due to the difficulties inherent in staging extranodal lymphomas (Blackledge *et al.*, 1979; Green *et al.*, 1981; Crowther *et al.*, 1982; Rao *et al.*, 1984; Bonadonna & Valagussa, 1986; Cajozzo *et al.*, 1987) by the Ann Arbor method, which the Manchester system was designed to circumvent. However no significant difference was found when either the Ann Arbor or the Manchester system was used, although it should be observed that the latter system was applied retrospectively. It is possible that the lack of any prognostic effect from stage in patients with gastric involvement and its strong prognostic effect found in patients with intestinal involvement may have been due to the different origins of lymphoid tissue, and hence histologic differences in the lymphomas arising in these two organs. However, treatment of patients in the series was preselected in respect of stage, with relatively aggressive treatment usually being given to patients with advanced stage, and less aggressive treatment given to those clinically localised disease. It is possible that this procedure effectively masked a prognostic effect of stage in patients with gastric involvement, though this remains unproven.

Patients received a wide range of treatments, depending on the BNLI protocols current at the time. Treatment groups available for comparison were small, and in many cases treatment was tailored to prognostic factors deemed to be important at the time, including histological grade, stage, and symptoms, thereby confounding the overall effects of therapy

**Figure 3** The overall survival of patients with (T) and without (NOT T) evidence of MHI.

and making statistically valid inferences hazardous. Thus the relatively high survival of patients who received complete surgical resection alone, compared to the survival of those who received CHOP or chlorambucil in addition, was probably due to the high proportion of patients in the former group with evidence of MALT type gastric lymphoma, since the survival of such patients was high in the series overall. In general, it would appear that survival depended primarily upon whether the extranodal involvement was of the stomach or of the intestine, the stage of the patient, whether the tumour originated from mucosa associated lymphoid tissue, and whether the tumour was a malignant histiocytosis of the intestine.

There is disagreement in the literature as to the need for surgical debulking. Those in favour of it argue that physical removal of as much tumour as possible reduces the risk of complications, such as haemorrhage or perforation, during subsequent chemotherapy or radiotherapy, as well as reducing the tumour load requiring treatment (Green *et al.*, 1981; Paulson *et al.*, 1983; Sheridan *et al.*, 1985; Bonadonna & Valagussa, 1986; Ampil, 1987; List *et al.*, 1988; Haber & Mayer, 1988; Baildam *et al.*, 1989); whilst other investigators have argued that this is not the case (Herrman *et al.*, 1980; Rao *et al.*, 1984; Cajozzo *et al.*, 1987; Gobbi *et al.*, 1990). In the present series two patients perforated, both after having undergone radical surgery followed by chemotherapy. However three patients who were only biopsied experienced fatal haematemesis and melaena during subsequent chemotherapy. In addition, there was one post-operative death which occurred following major surgery. These results suggest that surgical debulking does not necessarily protect the patients from complications. There is also disagreement as to whether adjuvant therapy following complete excision is necessary (Lim *et al.*, 1977; Herrmann *et al.*, 1980; Gospodarowicz *et al.*, 1983; Kaufman *et al.*, 1984; Rao *et al.*, 1984; Sheridan *et al.*, 1985; Bonadonna & Valagussa, 1986; Kajanti *et al.*, 1988; Jones *et al.*, 1988; Haber & Mayer, 1988; Mentzer *et al.*, 1988). It is of interest that in the present study, patients who received chemotherapy had a worrying incidence of side effects: of the 15 patients who died with infection, 12 were receiving chemotherapy at the time.

Individual centres see only small numbers of patients with GIT lymphoma over a long period, and there is therefore a need for a multicentre prospective study in order to create a database large enough for definitive analyses to be made in order to rationalise the treatment of such patients.

### References

AMPIL, F.L. (1987). Primary gastrointestinal lymphoma. *Oncology*, **44**, 214–218.

BAILDAM, A.D., WILLIAMS, G.T. & SCHOFIELD, P.F. (1989). Abdominal lymphoma–the place for surgery. *J. R. Soc. Med.*, **82**, 657–660.

BENNETT, M.H., FARRER-BROWN, G., HENRY, K. & JELLIFFE, A.M. (1974). Classification of non-Hodgkin's lymphomas. (letter) *Lancet*, **2**, 405–406.

BLACKLEDGE, G., BUSH, H., DODGE, O.G. & CROWTHER, D. (1979). A study of gastrointestinal lymphoma. *Clin. Oncol.*, **5**, 209–219.

BONADONNA, G., VALAUSSA, P. (1986). Should lymphomas of gastrointestinal tract be treated differently from other disease presentations? *Eur. J. Cancer Clin. Oncol.*, **22**, 1295–1299.

CAJOZZO, A., PERRICONE, R., ABBADESSA, V. & TOLOMEO, M. (1987). Primary gastrointestinal involvement in non-Hodgkin's lymphomas. *Acta. Haematol.* (Basel), **78** (suppl 1), 151–156.

CONTREARY, K., NANCE, F.C. & BECKER, W.F. (1980). Primary lymphoma of the gastrointestinal tract. *Ann. Surg.*, **191**, 593–598.

COX, D.R. (1972). Regression models and life tables. *J. Roy. Statist. Soc.* (Series B), **34**, 187–220.

CROWTHER, D. & RANKIN, E.M. (1982). Staging patients with non-Hodgkin's lymphoma. *Br. J. Haematol.*, **52**, 357–364.

DE LAS HERAS, M., NAVARRETE, A., BAS, A., PEREZ-RIGAL, V., ALONSO, J.D., GARCIA-SOLANO, J. & RAMOS, J. (1989). Gastric lymphomas of associated lymphoid tissue. *Ecco*, **5**, (abstr. 0–0368).

DIXON, M.F., WYATT, J.L., BURKE, D.A. & RATHBONE, B.J. (1988). Lymphocytic gastritis–Relationship to Campylobacter pylori infection. *J. Pathol.*, **154**, 125–132.

FREEMAN, C., BERG., J.W. & CUTLER, S.J. (1972). Occurrence and prognosis of extranodal lymphomas. *Cancer*, **29**, 252–260.

GOBBI, P.G., DIONIGI, P., BARBIERI, F., CORBELLA, F., BERTOLONI, D., GRIGNANI, G., JEMOS, V., PIERESCA, A. & ASCARI, E. (1990). The role of surgery in the multimodal treatment of primary gastric non-Hodgkin's lymphomas. A report of 76 cases and review of the literature. *Cancer*, **65**, 2528–2536.

GOSPODAROWICZ, M.K., BUSH, R.S., BROWN, T.C. & CHUA, T. (1983). Curability of gastrointestinal lymphoma with combined surgery and radiation. *Int. J. Radiat. Oncol. Biol. Phys.*, **9**, 3–9.

GOSPODAROWICZ, M.K., SUTCLIFFE, S.B., BROWN, T.C., CHUA, T., BUSH, R.S. (1987). Patterns of disease in localised extranodal lymphomas. *J. Clin. Oncol.*, **5**, 875–880.

GREEN, J.A., DAWSON, A.A., LESSELLS, A.M., DONALD, D. & MACHIN, D. (1981). Prognostic Factors in Gastrointestinal Lymphoma. *Clin. Oncol.*, **7**, 115–121.

GUPTA, S., PANT, G.A. & GUPTA, S. (1981). A Clinicopathological Study of Primary Gastrointestinal Lymphoma. *J. Surg. Oncol.*, **16**, 49–58.

HABER, D.A. & MAYER, R.J. (1988). Primary Gastrointestinal Lymphoma. *Semin. Oncol.*, **15**, 154–169.

HERRMANN, R., PANAHON, A.M., BARCOS, M.P., WALSH, D. & STUTZMAN, L. (1980). Gastrointestinal involvement in non-Hodgkin's lymphoma. *Cancer*, **46**, 215–222.

ISAACSON, P.G. & WRIGHT, D.H. (1978). Intestinal lymphoma associated with malabsorption. *Lancet*, **1**, 67–70.

ISAACSON, P.G. & WRIGHT, D.H. (1983). Malignant lymphoma of mucosa-associated tissue. A distinctive type of B-cell lymphoma. *Cancer*, **52**, 1410.

ISAACSON, P.G., MACLENNAN, K.A. & SUBBUSWAMY, S.G. (1984). Multiple lymphomatous polyposis of the gastrointestinal tract. *Histopathology*, **8**, 641–656.

ISAACSON, P.G. & WRIGHT, D.H. (1984). Extranodal malignant lymphoma arising from mucosa-associated lymphoid tissue. *Cancer*, **53**, 2515.

ISAACSON, P.G., O'CONNOR, N.T.J., SPENCER, J., BEVAN, D.H., CONNOLLY, C.E., KIRKHAM, N., POLLOCK, D.J., WAINSCOAT, J.S., STEIN, H. & MASON, D.Y. (1985). Malignant histiocytosis of the intestine: a T-cell tumour. *Lancet*, **11**, 688–691.

ISAACSON, P.G. & SPENCER, J. (1988). Malignant lymphoma of mucosa-associated lymphoid tissue. In *Malignant Lymphomas*, Habeshaw, J.A. & Lauder, I. (ed) pp. 179–200. Churchill Livingstone: Edinburgh.

ISAACSON, P.G. (1990). Lymphoma of mucosa-associated lymph tissue. *Histopathology*, **16**, 627–619.

ISHII, Y.U., TOKAMI, Y., YUOSA, H., TAKEI, T., KIKUCHI, K. (1984). Two distinct antigen systems in human B lymphocytes: identification of cell surface and intracellular antigens using monoclonal antibodies. *Clin. Exp. Immunol.*, **58**, 183–191.

JOHNSSON, A., BRUN, E., CAVALLIN-STAHL, E., AKERMAN, M. (1990). Primary Gastric Non-Hodgkin's Lymphomas. Does the concept of 'mucosa-associated lymphoma' have any clinical relevance? *4th International Conference on Malignant Lymphoma*, Lugano (abstr. 74).

JONES, R.E., WILLIS, S., INNES, D.J. & WANEBO, H.J. (1988). Primary gastric lymphoma. Problems in staging and management. *Am. J. surg.*, **155**, 118–122.

KAJANTI, M., KARKINEN-JAASKELAINEN, M. & RISSANEN, P. (1988). Primary gastrointestinal non-Hodgkin's lymphoma. A review of 36 cases. *Acta. Oncol.*, **27**, 51–55.

KAUFMAN, Z., ELIASHIV, A., SHPITZ, B., WITZ, M., GRIFFEL, B. & DINBAR, A. (1984). Primary gastrointestinal lymphoma. A Review of 21 cases. *J. Surg. Oncol.*, **26**, 17–21.

LIM, F.E., HARTMAN, A.S., TAN, E.G.C., CADY, B. & MEISSNER, W.A. (1977). Factors in the prognosis of gastric lymphoma. *Cancer*, **39**, 1715–1720.

LIST, A.F., GREER, J.P., COUSAR, J.C., STEIN, R.S., JOHNSON, D.H., REYNOLDS, V.H., GRECO, F.A., FLEXNER, J.M. & HANDE, K.R. (1988). Non-Hodgkin's lymphoma of the gastrointestinal tract: an analysis of clinical and pathologic features affecting outcome. *J. Clin. Oncol.*, **6**, 1125–1133.

LOEHR, W.J., MUJAHED, Z., ZAHN, F.D., GRAY, G.F. & THORBJARNARSON, B. (1969). Primary lymphoma of the gastrointestinal tract: a review of 100 cases. *Ann. Surg.*, **170**, 232–238.

MAKEPEACE, A.R., FERMONT, D.C. & BENNETT, M.H. (1987). Gastrointestinal non-Hodgkin's lymphoma. *Clin. Radiol.*, **38**, 609–614.

MASON, D.Y., CORDELL, J., BROWN, M., PALLESON, G., RALFKIAER, M., ROTHBARD, J., CRUMPLEN, M. & GATTER, K.C. (1989). Detection of cells in paraffin wax-embedded tissue using antibodies against a peptide sequence from the CD3 antigen. *J. Clin. Path.*, **42**, 1194–1200.

MASON, D.Y., COMANS-BITTER, W.M., CORDELL, J.L., VERHOEVEN, M.-A.J. & VAN DONGEN, J.J.M. (1990). Antibody L26 recognises an intracellular epitope on the B-cell-associated CD20 antigen. *Am. J. Pathol.*, **136**, 1215–1222.

MENTZER, S.J., OSTEEN, R.T., PAPPAS, T.N., ROSENTHAL, D.S., CANELLOS, G.P. & WILSON, R.E. (1988). Surgical therapy of localised abdominal non-Hodgkin's lymphomas. *Surgery*, **103**, 609–614.

NORTON, A.J., RAMSAY, A.D., SMITH, S.W., BEVERLY, P.C.L. & ISAACSON, P.G. (1986). Nonoclonal antibody (UCHLI) that recognises normal and neoplastic T-cells in routinely fixed tissue. *J. Clin. Path.*, **39**, 399–405.

NOVAK, S., CARAVEO, J., TROWBRIDGE, A.A., PETERSON, R.F. & WHITE, R.R. (1979). Primary lymphomas of the gastrointestinal tract. *South Med. J.*, **72**, 1154–1158.

PAULSON, S., SHEEHAN, R.G., STONE, M.J. & FRENKEL, E.P. (1983). Large cell lymphomas of the stomach: improved prognosis with complete resection of all intrinsic gastrointestinal disease. *J. Clin. Oncol.*, **1**, 263–269.

PETO, R., PIKE, M.C., ARMITAGE, P., BRESLOW, N.E., COX, D.R., HOWARD, S.V., MANTEL, N., MCPHERSON, K., PETO, J. & SMITH, P.G. (1971). Design and analysis of randomised clinical trials requiring prolonged observations of each patient: II. Analysis and examples. *Br. J. Cancer*, **35**, 1–39.

RAO, A.R., KAGAN, A.R., POTYK, D., NUSSBAUM, H., CHAN, P., HINTZ, B.L., WOLLIN, M. & RYOO, M.C. (1984). Management of gastrointestinal lymphoma. *Am. J. Clin. Oncol.*, **7**, 213–219.

SHERIDAN, W.P., MEDLEY, G. & BRODIE, G.M. (1985). Non-Hodgkin's lymphoma of the stomach: a prospective pilot study of surgery plus chemotherapy in early and advanced disease. *J. Clin. Oncol.*, **3** 495–500.

The Non Hodgkin's Lymphoma Pathologic Classification Project. National Cancer Institute: sponsored study of classifications of Non Hodgkin's Lymphomas: summary and description of a working Formulation for clinical usage. (1982). *Cancer*, **49**, 2112–2135.